# Effect of Moderate-Intense Training and Detraining on Glucose Metabolism, Lipid Profile, and Liver Enzymes in Male Wistar Rats: A Preclinical Randomized Study

**DOI:** 10.3390/nu15173820

**Published:** 2023-08-31

**Authors:** Hira Shakoor, Jaleel Kizhakkayil, Mariyam Khalid, Amar Mahgoub, Carine Platat

**Affiliations:** 1 Department of Nutrition and Health, College of Medicine and Health Sciences, United Arab Emirates University, Al Ain P.O. Box 15551, United Arab Emirates; 201890012@uaeu.ac.ae (H.S.);; 2 Department of Pharmacology, College of Medicine and Health Sciences, Khalifa University, Abu Dhabi P.O. Box 127788, United Arab Emirates; 3 Department of Pharmacology and Therapeutics, College of Medicine and Health Sciences, United Arab Emirates University, Al Ain P.O. Box 15551, United Arab Emirates

**Keywords:** endurance training, physical inactivity, glucose, metabolism, insulin, glycolysis, type 2 diabetes

## Abstract

Exercise training positively regulates glucose metabolism. This study investigated the impact of training and detraining on glucose metabolism, lipid profiles, and liver enzymes. Twenty-six rats completed an initial 4-week moderate-intense training (T0–T4). Then, the animals were randomly assigned to two groups at the end of week 4: AT4: detraining for 8 weeks; AT8: training for 8 weeks and 4-week detraining. Six animals were sacrificed at T0 and T4, four animals/group at T8, and three/group at T12. The study continued for 12 weeks, and all parameters were assessed at T0, T4, T8, and T12. IPGTT significantly improved after 4 weeks of training (*p* < 0.01) and was further reduced in AT8 at T8. In AT8, 8-week training significantly reduced total cholesterol at T4 and T12 vs. T0 (*p* < 0.05), LDL at T4, T8, and T12 vs. T0 (*p* < 0.01), ALP at T8, T12 vs. T0 (*p* < 0.01), and increased HDL at T8 and ALT at T8 and T12 vs. T0 (*p* < 0.05). Triglycerides and hexokinase activity increased significantly at T4 and T8 (*p* < 0.05) and then decreased at T12 in AT8. Pyruvate and glycogen increased at T12 in AT8 vs. AT4. Eight-week training improved LPL and ATGL expressions. Training positively modulated insulin, glucose metabolism, and lipid profiles, but detraining reduced the benefits associated with the initial training.

## 1. Introduction

Industrialization and digitalization in modern societies affect our way of living, working, and eating. This resulted in decreased practice of physical activity. According to the World Health Organization (WHO), 23% of adults and 81% of adolescents (11–17 years) are insufficiently active in the world [1]. Since our genome has been selected for the body to function optimally under daily physical exertion conditions, a lack of physical activity inevitably induces major physiological and metabolic disturbances, including altered glucose metabolism and lipid profile, and insulin resistance, potentially leading to type 2 diabetes [2,3].

Physical activity is a bodily movement that induces energy expenditure above the basal energy level. It is recommended that adults practice at least 150 min of moderate-intensity aerobic physical activity every week [4]. Physical activity is also associated with several health benefits. Independent of its direct effect on energy expenditure, physical activity modulates body composition, lipid profiles, glucose homeostasis, and insulin sensitivity [5]. Evidence shows that regular physical training reduces blood glucose levels, HbA1c, and insulin resistance, similar to insulin treatment [6]. In addition, regular physical training can positively modulate hepatic lipid metabolism and blood lipid profiles by increasing high-density lipoprotein (HDL) and decreasing triglyceride (TG) and low-density lipoprotein (LDL) levels [7,8]. Moreover, physical training significantly increases the levels of liver enzymes (aspartate transaminase (AST) and alanine aminotransferase (ALT)) [9,10].

The mechanism through which regular physical training regulates glucose metabolism involves the modulation of glycolytic enzymes such as phosphofructokinase (PFK) and hexokinase (HK). Evidence has shown that high-intensity exercise training increases phosphofructokinase (PFK) activity [11,12,13], whereas endurance training decreases PFK activity in humans [14,15]. In addition, endurance training results in a transient increase in hexokinase II (HKII) transcription and mRNA levels and lipoprotein lipase (LPL) in human skeletal muscle [16,17]. Moderate-intensity training in mice for 6 weeks significantly increased glycogen storage in the muscle (*p* < 0.05), but no significant difference was observed in pyruvate [18]. Another recent study showed that repetition of long-term training in elderly men could decrease maximal fat oxidation and significantly upregulate HKII, glucose transporter type-4 (GLUT-4), and adipose triglyceride lipase (ATGL) expression, which in turn increases the capacity of glucose transport and muscle lipolysis of endogenous fat [19]. 

In contrast, stopping physical training results in partial or total loss of these physiological and metabolic adaptations, known as detraining [20,21]. Animal and human studies have reported that detraining is characterized by decreased glucose tolerance and insulin action [22,23]. This was observed in trained rats after only one week of training cessation [22]. Evidence in humans has shown that training cessation negatively affects insulin sensitivity and blood lipid profiles [24,25]. Some studies have been conducted on bed rest, and this model has been used to investigate how physical inactivity leads to the progression of chronic disease in healthy individuals. It has been reported that bed rest reduces insulin sensitivity and increases insulin resistance [26,27,28,29,30]. Additionally, evidence has shown that bed rest for 10–14 days enhances glucose intolerance and hyperinsulinemia following an oral glucose tolerance test (OGTT) [27,28,31]. Further, a study in male runners reported reduced HDL levels and post-heparin lipoprotein lipase activity with no change in total cholesterol, triglycerides, and LDL after 14–22 days of no physical activity [32]. Additionally, both continuous and interval training for 12 weeks followed by 4 weeks without training significantly reduced liver enzymes (ALT, AST, and alkaline phosphatase (ALP) in rats, compared to weeks 6 and 12 [33]. A study performed by Simoneau et al. did not report any impact on PFK after training cessation [13]. However, the impact of moderate-intensity training and detraining on glycolytic pathways still needs further exploration. 

Indeed, training cessation has a negative impact on the body and leads to the progression of chronic diseases, including type 2 diabetes. Most adaptations gained during endurance training can be lost after detraining. Transitioning to sedentary behavior or physical inactivity would disrupt glucose metabolism. This is justified by Wendorf and Goldfine, who stated that human tribal people (Pima Indians) [34] and animals (spiny mice and the Egyptian sand rats) do not have type 2 diabetes when they are in their native state; however, when exposed to a sedentary environment with a constant food supply, they develop type 2 diabetes [35]. This shows that the human body must be involved in regular physical activity to function properly and prevent disease.

The lifestyle of our ancestors was challenging and, thus, physically demanding. Being physically active was mandatory for hunting, searching for food, and protecting against predators [36,37]. The body’s functions have adapted to this daily metabolic stress. Due to advancements in technology, humans have evolved in an environment that demands less physical activity. This behavior likely outpaced the genome’s ability to adapt, leading to a disconnect between physiological and metabolic functions and environmental factors [38]. The fact is that human genetic makeup has remained relatively identical since the appearance of the ancestral Homo sapiens 40,000 years ago [39]. This underscores the concept that our genome has not been optimized for a lifestyle characterized by inactivity and sedentarity, enforcing the idea. This mismatch resulted in the development of multiple chronic metabolic disorders, such as insulin resistance, leading to diseases like diabetes and cardiovascular disease [38]. To gain deeper insights into the development of these chronic metabolic disorders, it is critical to closely examine the transition towards inactivity. This scrutiny aims to uncover novel avenues for preventative and therapeutic interventions. Compelling evidence has demonstrated different physiological and metabolic functions between active and inactive states, accentuating the importance of comprehending the mechanisms of this transition [40,41,42]. As such, it becomes increasingly vital to delve into the intricacies of this shift. By comparing individuals who stopped being physically active with those who are regularly trained, this objective can be achieved. This comparison will help to pinpoint the most effective method of reintroducing physical activity to individuals who lead sedentary lives but are genetically programmed for a physically active life. Building on such a design, the current study seeks to investigate the changes in metabolic processes that occur as the body transitions from an active lifestyle to a state of physical inactivity. Additionally, this study also investigated the impact of physical training and detraining on insulin, glucose tolerance, lipid profiles, liver enzymes, and the glycolytic pathway. 

## 2. Materials and Methods

### 2.1. Ethical Approval and Animal Care

The ethical approval for the animal study was obtained from the Animal Research Ethics Committee of the College of Medicine and Health Sciences at UAE University (ERA_2021_7238). The use and care of all animals in the experiment were according to the safe practice for animals in research guidelines as stipulated by the National Institutes of Health, USA.

Rats were maintained in an air-conditioned room (22 ± 2 °C) with a 12 h light/12 h dark cycle and 50% humidity. All animals received the same standard feed containing 24% protein, 5% fiber, and 8% ash.

### 2.2. Sample Size Calculation

The sample size was determined according to the estimation obtained from Mead’s Resource Equation [43]. From this equation, two to three animals are required per group. Therefore, the final required sample size was equal to 26 (=6 + 6 + 7 + 7). 

### 2.3. Training and Experimental Protocol

Twenty-six adult male Wistar rats (4–6 weeks old) were used in this study and bred in-house at the Animal Research Facility at the College of Medicine and Health Sciences at UAE University. The total duration of the study was 12 weeks. 

At baseline (T0), six rats were sacrificed by decapitation. The remaining 20 rats underwent a 4-week exercise training program on a treadmill (6 Lanes; Columbus Instruments Treadmill Simplex II, Columbus, OH, USA). Each day during the first week, the protocol added 1 m/min to familiarize rats with the treadmill training. For the training protocol for 4 weeks, the treadmill speed was set to 5 m/min on the first day for 5 min per day. At the beginning of the third week, the treadmill speed reached 25 m/min. The 20-min duration was increased every week until it reached 60 min. After that, the speed and duration of running were kept constant. The treadmill was set at a 0% slope. Rats who stopped occasionally during running were gently prodded to help them continue running, and if required only, slight electrical stimuli (0.2 mA) were given. 

**Figure 1:**
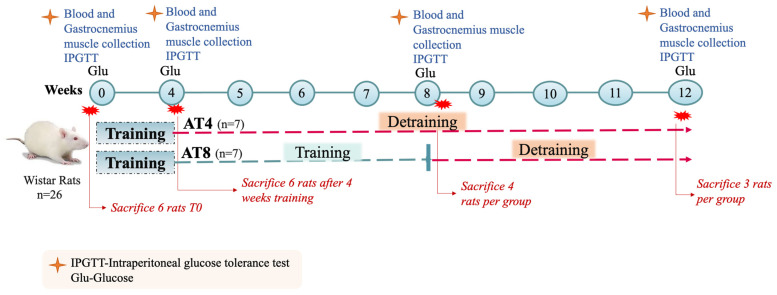
Study protocol. Aerobic training of 4 weeks (AT4), aerobic training of 8 weeks (AT8).

After 4 weeks of training (T4) and 48 h after the last exercise session, to avoid any acute effect of exercise, 6 rats were sacrificed. The other remaining 14 animals were randomly divided into two groups: (1) training cessation till week 12 (group AT4); (2) aerobic training for 4 additional weeks, on the treadmill at 25 m/min for 1 h per day, 5 days per week, followed by 4 weeks with no training (Group AT8). Four animals per group were sacrificed at T8, and all remaining animals (n = 6) were sacrificed at T12 (Figure 1).

### 2.4. Blood Collection

Blood was collected immediately after each sacrifice at different times (T0, T4, T8, and T12). Blood samples were drawn into a dry and sterile Vacutainer for a serum with a gel clot activator. Blood samples were centrifuged at 1500× *g* for 20 min, and serum was collected and stored at −80 °C until analyzed. 

### 2.5. Feed Intake, Body Weight, and Blood Glucose

During the 12-week study period, feed intake and body weight were measured at T0, T4, T8, and T12.

### 2.6. Intraperitoneal Glucose Tolerance Test (IPGTT)

The intraperitoneal glucose tolerance test (IPGTT) was performed at baseline (T0), 4 weeks (T4), 8 weeks (T8), and 12 weeks (T12). Animals were fasting for 10 h, and each conscious rat was intraperitoneally injected (2 g/kg body weight). For glucose solution preparation, 30 g was dissolved in 40 mL of distilled H_2_O. Fasting (0 min) and stimulated blood glucose levels were measured 30, 60, 120, and 180 min after glucose administration. Blood was collected from the tail. The glucose area under the curve (AUC) was calculated based on individual animals’ fasting blood glucose levels over time using GraphPad Prism 9.0 (GraphPad, San Diego, CA, USA).

### 2.7. Lipid Profile, Glucose, and Liver Enzyme

Glucose, total cholesterol, low-density lipoprotein (LDL), high-density lipoprotein (HDL), triglycerides, aspartate transaminase (AST), alanine transaminase (ALT), and alkaline phosphatase (ALP) were measured in serum using enzymatic colorimetric methods on Roche/Hitachi Cobas C systems (Integra 400 Plus, Mannheim, Germany).

### 2.8. Serum Insulin

Insulin was assessed using a commercially available ELISA kit, Merck Millipore (Cat# EZRMI-13K), and analysis was performed based on the manufacturer’s instructions.

### 2.9. Enzymatic Activity

Following the manufacturer’s instructions, gastrocnemius muscle tissues were homogenized in an ice-cooled assay buffer and centrifuged at 13,000× *g* at 4 °C for 15 min. Phosphofructokinase (PFK) and hexokinase (HK) enzyme activity was measured immediately in the supernatant using a commercially available PFK activity assay kit (ab155898, Abcam, Cambridge, UK) and HK activity assay kit (ab136957, Abcam, Cambridge, UK).

### 2.10. Muscle Glycogen and Pyruvate

A small portion of the gastrocnemius muscle (10 mg) was homogenized in 200 μL of ice-cold water (ddH_2_O) using a pestle on ice. Homogenates were then boiled for 10 min at 100 °C to inactivate enzymes in the sample and centrifuged for 10 min at 13,000× *g* at 4 °C. Glycogen was determined in the supernatant using a glycogen assay kit (ab65620, Abcam, Cambridge, UK).

The gastrocnemius muscle (10 mg) was homogenized in 500 μL of a pyruvate assay buffer using a pestle kept on ice and centrifuged for 15 min at 13,000× *g* at 4 °C. Pyruvate was determined in the supernatant using a pyruvate assay kit (ab65342, Abcam, Cambridge, UK).

### 2.11. Lactate

In AT4, blood lactate concentration was measured at T4 and T12. In AT8, lactate was measured at T4, T8, and T12 by a Lactate Plus handheld blood lactate meter (Nova Biomedical, Waltham, MA, USA). One drop of blood was collected via tail vein puncture onto a disposable strip for lactate analysis. Before measurements in the resting condition, all rats were physically restrained for 30 min in small plastic containers to ensure that all rats were as physically inactive as possible. Lactate was measured at rest and after every 5 min while rats ran on a treadmill at 25 m/min speed for 25 min.

### 2.12. Immunoblot Analysis

The lipoprotein lipase (LPL), adipose triglyceride lipase (ATGL), and fatty acid transport protein 4 (FATP4) expressions were evaluated in skeletal muscle homogenates. Total protein was isolated from the gastrocnemius muscle using a RIPA lysis buffer (Sigma Aldrich, St. Louis, MO, USA) containing a 1% protease phosphatase inhibitor cocktail (Sigma Aldrich, St. Louis, MO, USA) using a homogenizer. The total protein in the supernatant was determined by the BCA Protein Assay Kit (Sigma Aldrich, St. Louis, MO, USA). Then, it was diluted, the supernatant was diluted in a 6× RIPA buffer and heated for 5 min at 90 °C.

For electrophoresis, 35 μg of protein from each sample was loaded per well and separated by SDS–PAGE. The protein was transferred onto a nitrocellulose membrane by wet transfer using a Bio-Rad Electro-transfer apparatus. Membranes were blocked with 5% skimmed milk in tris-buffered saline with 0.1% Tween-20 (TBS-T) at room temperature for 1 h. Then, membranes were incubated overnight with primary antibodies, including FATP4 and LPL antibodies (Abcam, Cambridge, UK), ATGL antibody (Cell Signaling Technology, Danvers, MA, USA), and GAPDH (ABclonal, Woburn, MA, USA). The appropriate horseradish peroxidase-conjugated secondary antibodies (Jackson Immune Research, Cambridge House, UK) were used to blot for 1 h after being washed three times with TBS-T. The band densities were detected using an enhanced chemiluminescence detection kit (Bio-Rad, Hercules, CA, USA). The band densities were quantified by the image analyzer Quantity One System (Bio-Rad, Hercules, CA, USA).

### 2.13. Statistical Analysis

Statistical analysis was performed using the SPSS software (v.28). A paired *t*-test was performed to analyze the intraperitoneal glucose tolerance test (IPGTT) at T0 and T4. An independent *t*-test was conducted to analyze the difference between groups at T8 and T12. To assess the normality assumptions of the data, Shapiro–Wilk’s test was applied, and it was found that five variables (cholesterol, ALP, AST, ALT, and pyruvate) violated the normality assumptions. The non-parametric Kruskal–Wallis test was employed for these variables, and subsequent multiple comparisons were performed using Dunn’s test. For non-parametric tests, data are expressed as median. A mixed model (two-way analysis of variance (ANOVA) with a Sidak Post Hoc test pairwise comparisons was performed to compare groups and time points. All experiments were carried out in duplicates. Data are expressed as means ± standard deviation (S.D.). In all analyses, *p* ≤ 0.05 was considered statistically significant (* *p* ≤ 0.05, ** *p* ≤ 0.01).

## 3. Results

### 3.1. Body Weight

The mean weight at baseline (T0) was similar in AT4 (170.03 ± 20.80 g) and AT8 (173.77 ± 27.71 g) (*p* < 0.05) (Table 1). After 4 weeks of training, the weight significantly increased to 258.05 ± 17.34 and 258.25 ± 13.63 in AT4 and AT8, respectively (*p* < 10^−4^). In both AT4 and AT8, the body weight further significantly increased at T8 (324.30 ± 36.34 g and 330.16 ± 22.26 g, respectively) and T12 (351.40 ± 46.86 g and 356.57 ± 8.86 g, respectively) compared to T0 and T4 (*p* ≤ 0.01). However, in AT8, the body weight was greater than the AT4 group at both T8 (*p* = 0.689) and T12 (*p* = 0.961) but did not reach statistically significant.

### 3.2. Feed Intake

Up to T4, the two groups of animals were not physically separated into different cages. The average daily feed intake of the rats at baseline was 24.35 ± 3.18 g, slightly increasing to 25.86 ± 4.47 g at T4. In AT4, feed intake decreased to 23.73 ± 2.30 g in T8 after exercise cessation, then 21.41 ± 1.33 g at T12. In AT8, the feed intake was lower at T8 (23.39 ± 4.43 g) and remained the same at T12. None of the differences were significant (Table 1).

### 3.3. Intraperitoneal Glucose Tolerance Test (IPGTT)

**Figure 2:**
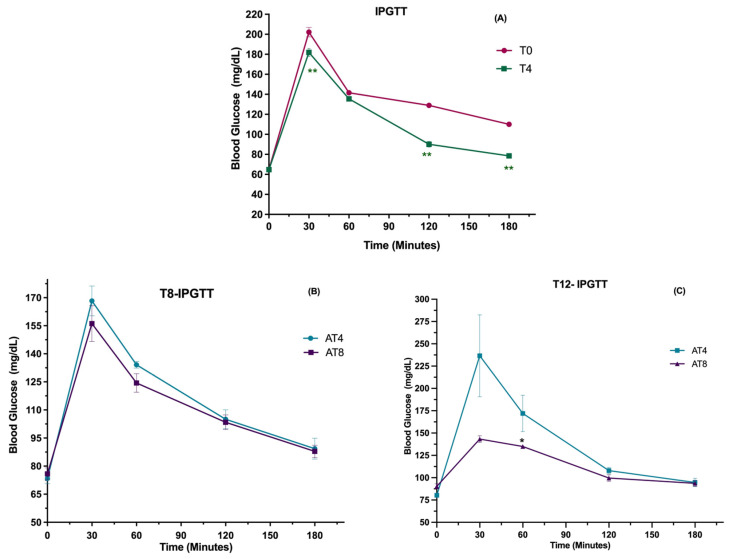
Intraperitoneal glucose tolerance test (IPGTT). (**A**) A paired *t*-test was performed to analyze the IPGTT at time points T0 and T4. (**B**) An independent *t*-test was performed to analyze the difference between AT4 and AT8 at T8. (**C**) An independent *t*-test was performed to analyze the difference between AT4 and AT8 at T12. Data are shown as Mean ± S.D. Statistical significance was set at *p* ≤ 0.05. Statistically significant * (*p* ≤ 0.05) and ** (*p* ≤ 0.01).

The total area under the curve (AUC) of IPGTT decreased significantly by 32% from 12,742 ± 1113 at T0 to 8580 ± 1132 at T4 as a result of 4 weeks of training (*p* < 0.001) (Figure 2A). In AT4, after 4 weeks without physical activity (T8), the AUC increased to 8873 ± 327 and even more at T12 (11,865 ± 2095). Inversely, the glucose AUC continued decreasing in AT8, at T8 (7971 ± 331), after 4 more weeks of training, and at T12 (7020 ± 371), after 4-week detraining (Figure 2B). At T12, even after 4 weeks of detraining, the glucose peak significantly decreased in AT8 (*p* = 0.046) at 60 min compared to AT4 (Figure 2C). 

### 3.4. Serum Glucose

**Figure 3:**
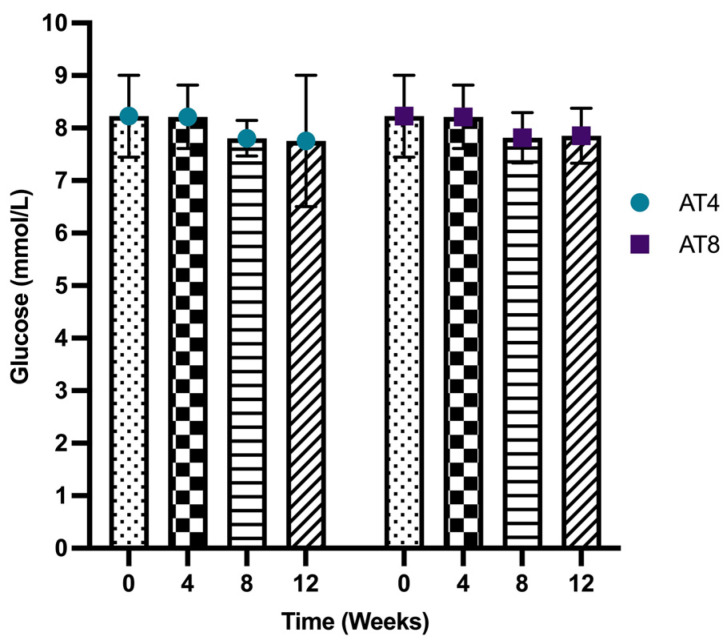
Serum glucose level. Data are shown as Mean ± S.D. of duplicate experiments. ANOVA with multiple comparisons was performed, and statistical significance was set at *p* ≤ 0.05.

Serum glucose level was not significantly different between T0 and T4 (8.23 ± 0.78 mmol/L and 8.21 ± 0.61 mmol/L, respectively). In both groups, serum glucose levels did not change significantly over time. In addition, no change was observed in glucose levels between AT4 and AT8 groups neither at T8 nor at T12 (Figure 3).

### 3.5. Serum Insulin

**Figure 4:**
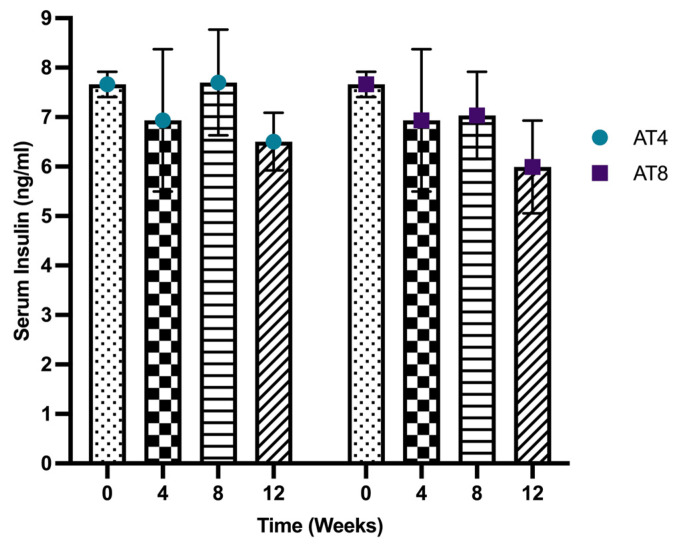
Serum insulin level. Data are shown as Mean ± S.D. of duplicate experiments. ANOVA with multiple comparisons was performed, and statistical significance was set at *p* ≤ 0.05.

After 4 weeks of moderate-intense training, the serum insulin level decreased from 7.66 ± 0.26 ng/mL to 6.93 ±1.44 ng/mL (Figure 4). In AT4, the serum insulin level increased to 7.67 ± 1.07 ng/mL after 4 weeks of detraining at T8, then decreased again at T12, but it was not significant. In AT8, the insulin level (7.03 ± 0.88 ng/mL) at T8 remained similar to T4. However, after 4 weeks of detraining, at T12, the insulin level was lowered (5.99 ± 0.94 ng/mL). Compared to AT4, the serum insulin level was lower in the AT8 group at T8 and T12, but this did not reach statistical significance.

### 3.6. Serum Lipid Profiles

#### 3.6.1. Serum Total Cholesterol Concentration

**Figure 5:**
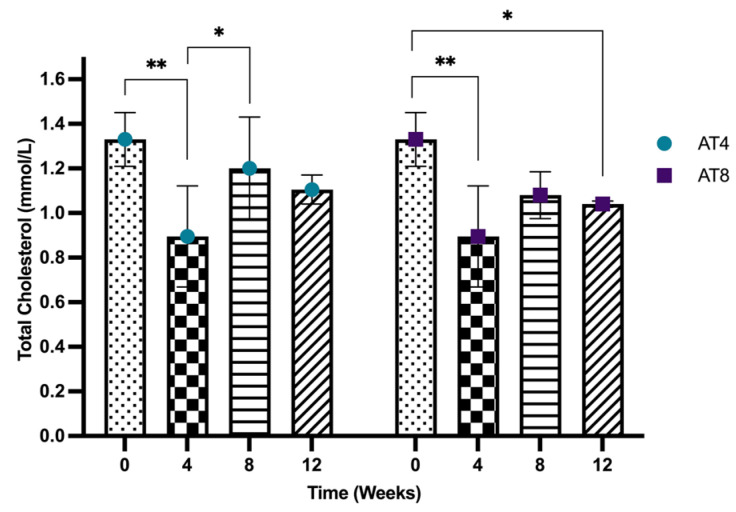
Serum cholesterol concentration. Data are shown as Median, 95% CI of duplicate experiments. A Kruskal–Wallis test with multiple comparisons of Dunn’s test was performed, and statistical significance was set at *p* ≤ 0.05. Statistically significant difference among time points * (*p* ≤ 0.05) and ** (*p* ≤ 0.01).

The serum total cholesterol concentration reduced significantly from 1.33 mmol/L, 95% CI at baseline, to 0.89 mmol/L, 95% CI after 4 weeks of training (*p* < 10^−4)^). In AT4, the cholesterol level significantly increased at T8 (1.20 mmol/L, 95% CI) after 4 weeks of detraining compared to T4 (*p* = 0.023), and no further significant change was observed at T12, while in AT8, even after 4 weeks of detraining, the serum cholesterol level at T12 (1.04 mmol, 95% CI) remained significantly lower than the level at T0 (*p* = 0.004). Cholesterol levels remained lower in the AT8 group at T8 and T12 compared to AT4, but there was no significant difference among the groups (Figure 5).

#### 3.6.2. Serum Triglycerides Concentration

**Figure 6:**
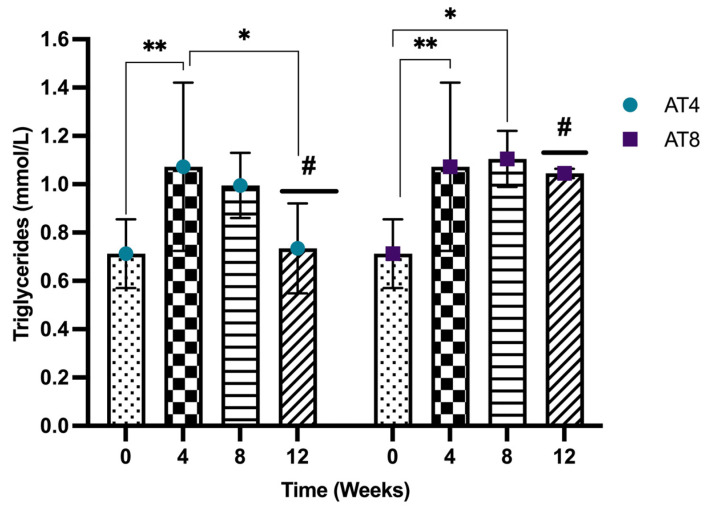
Serum triglycerides concentration. Data are shown as Mean ± S.D. of duplicate experiments. ANOVA with multiple comparisons was performed, and statistical significance was set at *p* ≤ 0.05. Statistically significant difference among time points * (*p* ≤ 0.05) and ** (*p* ≤ 0.01). Statistically significant difference among groups # (*p* ≤ 0.05).

The serum triglyceride concentration significantly increased from 0.71 ± 0.14 mmol/L at baseline to 1.07 ± 0.35 mmol/L after 4 weeks of training (*p* = 0.009). In AT4, after training cessation, the triglyceride level started to decrease to reach 0.99 ± 0.13 mmol/L at T8, then 0.73 ± 0.17 mmol/L at T12, which is significantly lower than the value at T4 value but still similar to the baseline level. In contrast, in AT8, triglyceride levels increased significantly to 1.10 ± 0.12 mmol/L (*p* = 0.021) at T8 compared to baseline, and a slight reduction after training cessation of 0.96 ± 0.02 mmol/L was observed at T12. Moreover, at T12, in AT4, the serum triglycerides level was significantly lower than AT8 (*p* = 0.011) (Figure 6).

#### 3.6.3. Serum Low-Density Lipoprotein (LDL) and High-Density Lipoprotein (HDL) Concentration

**Figure 7:**
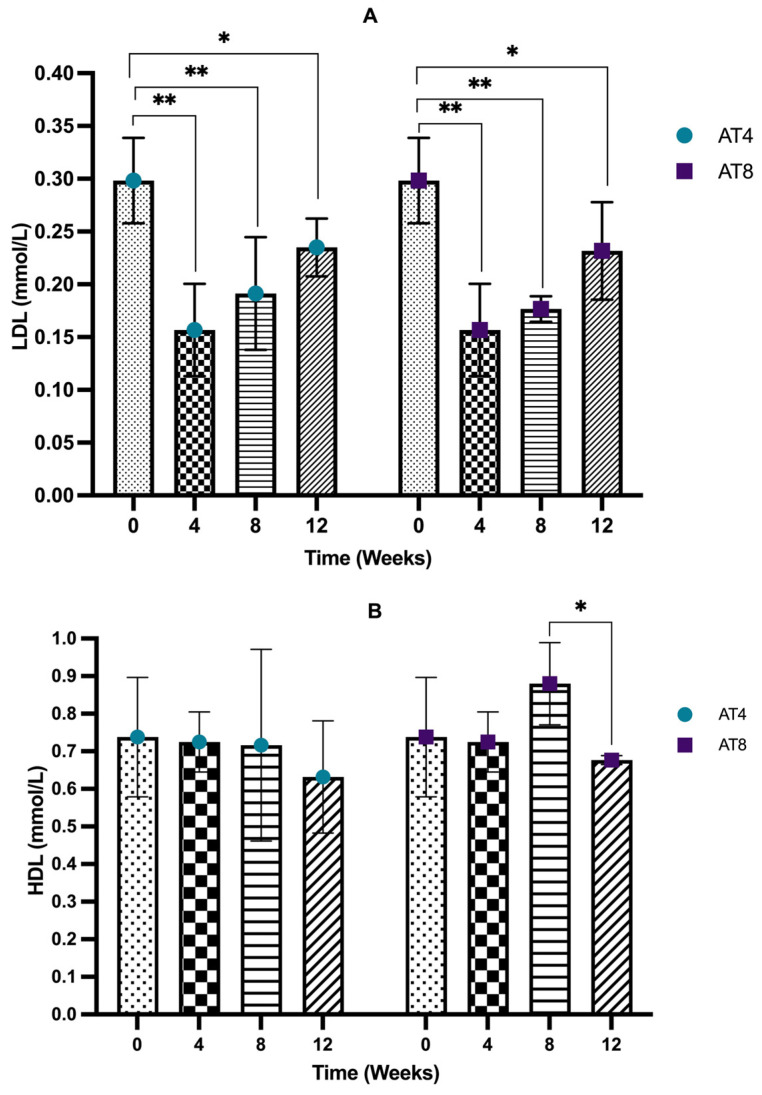
Serum low-density lipoprotein (LDL) and high-density lipoprotein (HDL) concentration. (**A**) LDL Comparison between time points and between groups. (**B**) HDL comparison between groups for each time point and between groups. ANOVA with multiple comparisons was performed, and statistical significance was set at *p* ≤ 0.05. Statistically significant difference among time points * (*p* ≤ 0.05) and ** (*p* ≤ 0.01).

The serum low-density lipoprotein (LDL) level decreased significantly (*p* < 10^−4^) from 0.30 ± 0.04 mmol/L at baseline to 0.16 ± 0.04 mmol/L after 4 weeks of training (Figure 7A). An increasing LDL trend was observed in AT4 after detraining. However, LDL level at both T8 and T12 remains significantly lower than baseline (*p* < 0.05). A similar trend was observed in AT8; after detraining at T12, the serum LDL level increased. 

The serum high-density lipoprotein (HDL) level was maintained at T4 (0.72 ± 0.08 mmol/L) compared to baseline (0.74 ± 0.16 mmol/L). In AT4, no change was observed at T8. Only a slight decrease was noted at T12, but it remained insignificant (Figure 7B). Interestingly, in AT8, 8 weeks of training (T8) significantly increased the HDL level to 0.88 ± 0.11 mmol/L (*p* = 0.009), while after 4 weeks of detraining (T12), the HDL level decreased significantly to 0.68 ± 0.01 mmol/L (*p* = 0.013). No significant difference was observed between the two groups.

### 3.7. Serum Liver Enzymes

**Figure 8:**
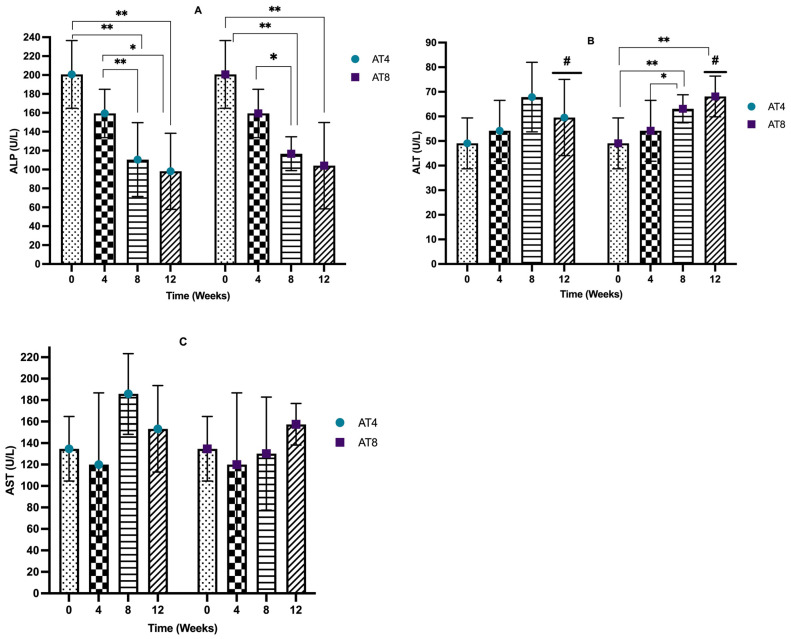
Serum liver enzymes. (**A**) Alkaline phosphatase (ALP) level comparison between time points and between groups. (**B**) Serum alanine transaminase (ALT) comparison between time points and between groups. (**C**) Aspartate aminotransferase (AST) comparison between time points and between groups. Data are shown as Median, 95% CI of duplicate experiments. A Kruskal–Wallis test with multiple comparisons of Dunn’s test was performed, and statistical significance was set at *p* ≤ 0.05. Statistically significant difference among time points * (*p* ≤ 0.05) and ** (*p* ≤ 0.01). Statistically significant difference among groups # (*p* ≤ 0.05).

The serum alkaline phosphatase (ALP) level decreased after 4 weeks of training from 200.7 U/L, 95% CI at T0 to 159.35 U/L, 95% CI at T4, but it is not significant. In AT4, after training cessation, the ALP level decreased drastically to a lower level of 110.5 U/L (95% CI, *p* < 10^−4^) at T8 compared to T0. Then, it decreases to 98.15 U/L, 95% CI at T12, which is significantly lower than the baseline and T4 values (*p* = 0.01). The ALP level significantly (*p* ≤ 0.02) decreased in AT8 to 116.75 U/L, 95% CI at T8 compared to T0 and T4. Then, in AT8, there was a decreasing trend in the ALP level after training cessation (104.1 U/L, 95% CI) at T12; the value is significantly (*p* = 0.002) lower than the baseline value. No significant difference was observed between AT4 and AT8 (Figure 8A).

The serum alanine phosphatase (ALT) level was not altered significantly after 4 weeks of training. In AT4, 4 weeks of detraining elevated the ALT level (67.85 U/L, 95% CI) at T8. A similar trend was observed in AT8; after 4 weeks of detraining, the ALT level increased to 68.1 U/L, 95% CI, *p* = 0.001 at T12. AT4 and AT8 significantly differed at T8 (*p* = 0.042) (Figure 8B).

The serum aspartate aminotransferase (AST) level decreased from 134.65 U/L, 95% CI at baseline 119.95 U/L, and 95% CI at T4, but it was not significant. In AT4, the AST level abruptly increased to 185.8 U/L, 95% CI at T8 after training cessation, then decreased to 153.3 U/L, 95% CI at T12. In AT8, the AST level was maintained at T8 (130.15 U/L, 95% CI) and then increased at T12 (157.5 U/L, 95% CI) after training cessation. No significant difference was seen between the AT4 and AT8 (Figure 8C).

### 3.8. Phosphofructokinase (PFK)

**Figure 9:**
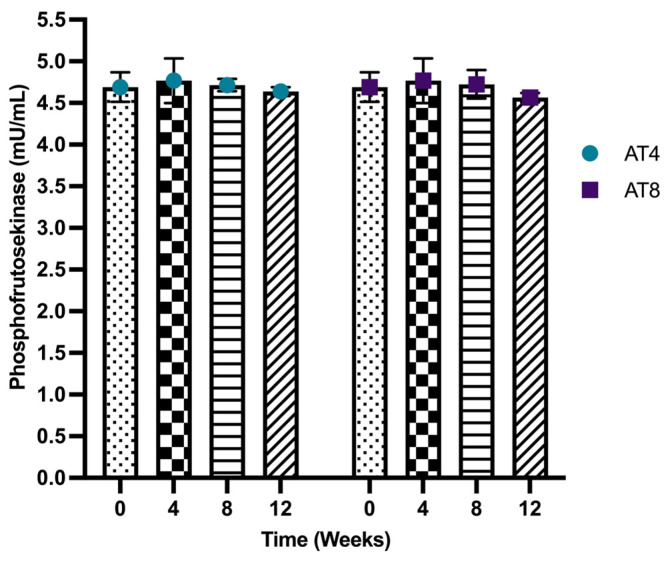
Phosphofructokinase (PFK) level in gastrocnemius muscle. Data are shown as Mean ± S.D. of duplicate experiments. ANOVA with multiple comparisons was performed, and statistical significance was set at *p* ≤ 0.05.

Phosphofructokinases (PFK) were assessed in the gastrocnemius muscle of rats. The PFK level was 4.69 ± 0.17 mU/mL at baseline and 4.77 ± 0.27 mU/mL at T4. The PFK level was maintained in AT4 and AT8 at all time points. No significant change was observed between different time points or between groups (Figure 9).

### 3.9. Hexokinase (HK)

**Figure 10:**
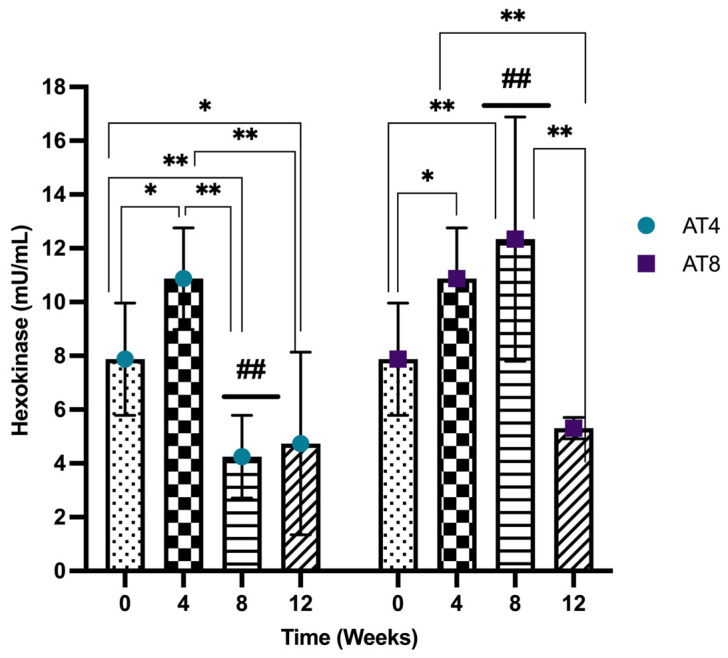
Hexokinase (HK) level in gastrocnemius muscle. Data are shown as Mean ± S.D. of duplicate experiments. ANOVA with multiple comparisons was performed, and statistical significance was set at *p* ≤ 0.05. Statistically significant difference among time points * (*p* ≤ 0.05) and ** (*p* ≤ 0.01). Statistically significant difference among groups ## (*p* ≤ 0.01).

Hexokinase (HK) activity was assessed in the gastrocnemius muscle of rats, and it increased significantly after 4 weeks of training from 7.87 ± 2.09 mU/mL at T0 and 10.87 ± 1.89 mU/mL at T4 (*p* ≤ 0.05). However, in AT4, after 4 weeks of detraining, HK decreased at T8 (4.25 ± 1.54 mU/mL) and was significantly lower than baseline and T4 values (*p* < 10^−4^). No further change was observed at T12 compared to T8. In AT8, the HK continued to increase with 8 weeks of training (12.34 ± 4.54 mU/mL compared to baseline, *p* = 0.004). Then, it decreased drastically to 5.31 ± 0.39 mU/mL at T12 after 4 weeks of detraining (*p* < 10^−4^). At T8, there was a significant (*p* < 10^−4^) difference observed among AT4 (4 weeks of detraining) and AT8 (continuous training for 8 weeks) (Figure 10).

### 3.10. Pyruvate

**Figure 11:**
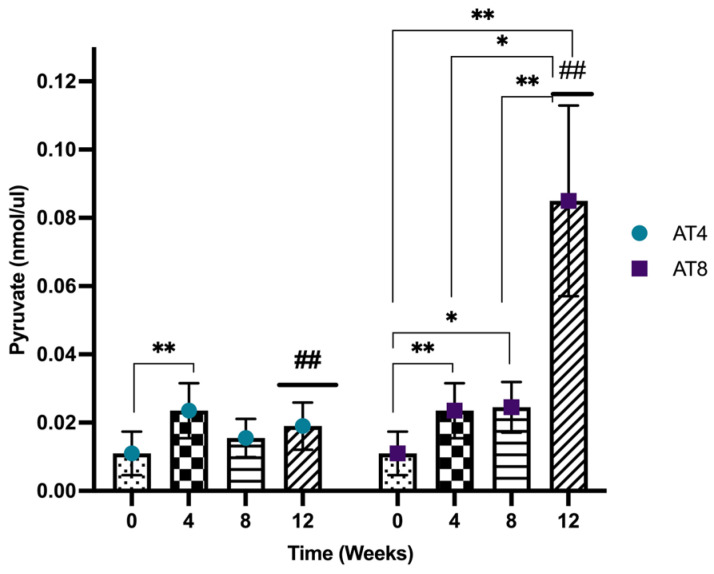
Pyruvate level in gastrocnemius muscle. Data are shown as the Median of duplicate experiments. Kruskal–Willis test with multiple comparisons Dunn’s test was performed, and statistical significance was set at *p* ≤ 0.05. Statistically significant difference among time points * (*p* ≤ 0.05) and ** (*p* ≤ 0.01). Statistically significant difference among groups ## (*p* ≤ 0.01).

Pyruvate was assessed in the gastrocnemius muscle of rats, and it increased significantly after 4 weeks of training from 0.01 nmol/uL, 95%CI at baseline and 0.02 nmol/uL, 95% CI at T4 (*p* = 0.002). In AT4, the pyruvate level was then maintained at a similar level at T8 and T12 after training cessation. In AT8, the pyruvate level was maintained at T8 (*p* = 0.02) but increased significantly to 0.08 nmol/uL, 95% CI, *p* < 10^−4^ at T12 after 4 weeks of training cessation compared to baseline, T4 and T8 (*p* ≤ 0.01). A significant difference between AT4 and AT8 was observed at T12 (*p* = 0.042) (Figure 11).

### 3.11. Glycogen

**Figure 12:**
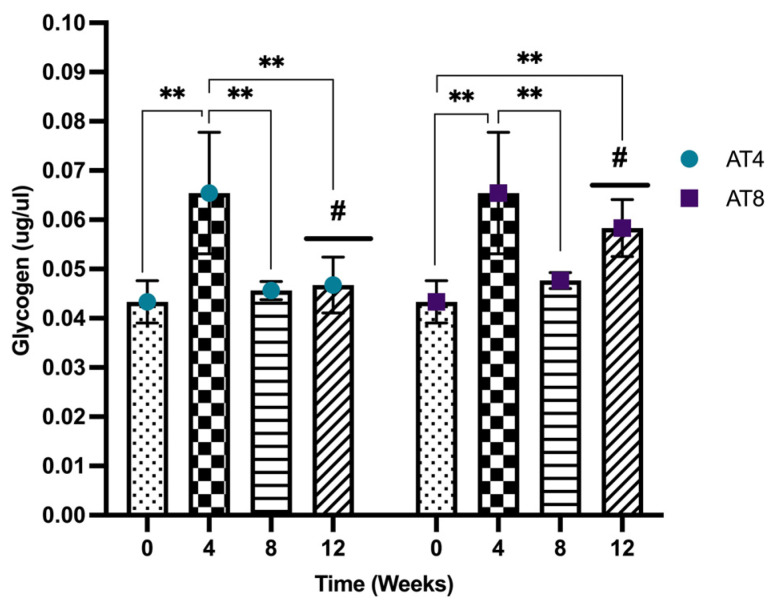
Glycogen level in gastrocnemius muscle. Data are shown as Mean ± S.D. of duplicate experiments. ANOVA with multiple comparisons was performed, and statistical significance was set at *p* ≤ 0.05. Statistically significant difference among time points ** (*p* ≤ 0.01). Statistically significant difference among groups # (*p* ≤ 0.05).

Glycogen increased significantly after 4 weeks of training from baseline (0.04 ± 0.00 ug/uL) to T4 (0.06 ± 0.01 ug/uL) (*p* < 10^−4^). Then, the glycogen concentration decreased significantly in both groups (AT4 and AT8) at T8 (*p* ≤ 0.01). In AT4, no further decrease was observed at T12, but the level remained lower than T4 (*p* = 0.002). In AT8, the glycogen level tended to increase at T12 after 4 weeks of training cessation and was higher than the baseline level (*p* = 0.004). At T12, the glycogen level was higher in AT8 compared to AT4 (*p* = 0.16) (Figure 12).

### 3.12. Lactate

**Figure 13:**
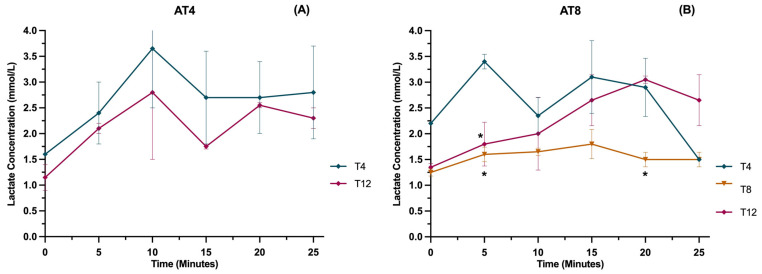
Blood lactate level in the AT8 group. (**A**) Lactate concentration in the AT4 group and (**B**) lactate concentration in the AT4 group. Data are shown as Mean ± S.D. of duplicate experiments. ANOVA with multiple comparisons was performed, and statistical significance was set at *p* ≤ 0.05. Statistically significant difference among time points * (*p* ≤ 0.05).

The lactate level was measured in running rats at various time points (0 min, 5 min, 10 min, 15 min, and 25 min) at a velocity of 25 m/min (Figure 13A). In AT4, overall values were lower at T12 compared to T4 after 8 weeks of detraining, but the results were not statistically significant. In AT8, lactate values were lower at T8 than T4, while at T12, values were higher, especially after 15 min of running at 25 m/min. There is a significant difference in the AT8 group at 5 min and 20 min between T8 and T4 (*p* ≤ 0.05) (Figure 13B).

### 3.13. Protein Expressions

**Figure 14:**
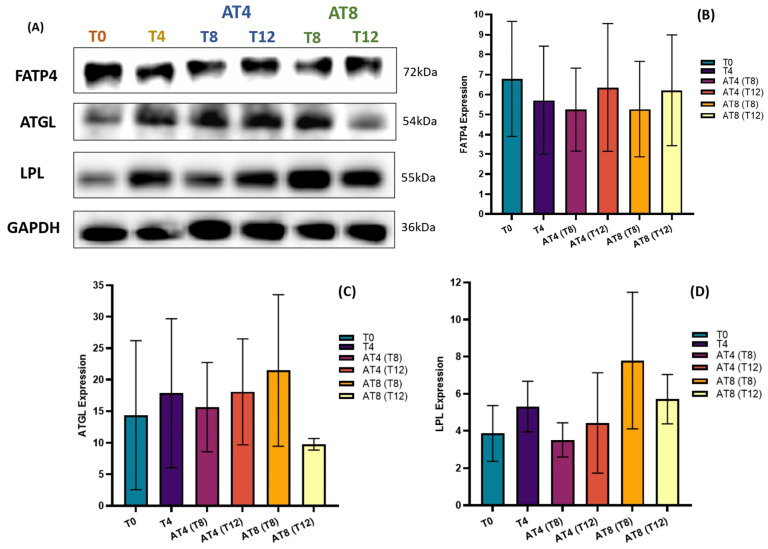
Effect of training and detraining on protein expression. (**A**) Immunoblot analysis, (**B**) level of FATP4, expression (**C**) level of ATGL expression, and (**D**) level of LPL expression. Data are shown as Mean ± S.D. of duplicate experiments. ANOVA with multiple comparisons was performed, and statistical significance was set at *p* ≤ 0.05.

The level of protein expression of FATP4, ATGL, and LPL was assessed by Western blotting in the rat’s gastrocnemius muscle (Figure 14A). Training slightly reduced the FATP4 protein expression, but the change was not statistically significant (Figure 14B). However, 4 weeks of detraining in AT4 slightly decreased FATP4 expression, but after 8 weeks, it increased by 11% ± 3.19. In AT8, 4 weeks of detraining at T12, increased FATP4 expression by 18% ± 2.77.

There was an increase in ATGL by 24% ± 7.07 at T4 compared to baseline. In AT4, after 4 weeks of detraining, AGTL decreased by 12% ± 7.07 at T8, but at T12, the expression level remained similar to T4. In AT8, ATGL increased by 37% ± 12.02 at T8 with 8 weeks of training but then decreased by 54% ± 0.92 at T12 (Figure 14C). 

There was an increase in LPL by 37% ± 0.91 at T4 compared to T0. In AT4, after 4 weeks of detraining, LPL expression decreased by 33% ± 0.91 at T8 and 16% ± 2.69 at T12 compared to T4. In AT8, LPL increased by 121% ± 3.68 at T8 with 8 weeks training, then decreased by 26% ± 1.32 at T12 after detraining (Figure 14D). 

## 4. Discussion

Physical activity is the naturally programmed status of the body. The present study reported the metabolic and biochemical adaptations to regular endurance training and the alteration in the metabolic process and glucose homeostasis when the body switched from training to detraining. In addition, the dose effect of moderately intense aerobic training and detraining from short duration (4 weeks) to long duration (8 weeks) was considered. Regular moderate-intense aerobic training positively regulates blood glucose, insulin levels, glucose tolerance, lipid profile, and glycolytic enzymes, but training cessation disrupts glucose homeostasis. 

Increased physical activity is a clinically proven first-line strategy to improve glucose metabolism. The current study reported the beneficial effect of moderate-intensity aerobic training on glucose tolerance lost during the detraining period. The AUC of IPGTT was 11% greater in AT4 after training cessation compared to the AT8 group at T8. When the detraining continued for 8 weeks (T12) in the AT4 group, the AUC of IPGTT increased by 34% compared to T8. Previous findings reported that healthy individuals who underwent bed rest for 1–3 weeks showed abnormal intravenous glucose tolerance tests [44]. On the other hand, the AT8 group who continued exercise training for 8 weeks had improved glucose tolerance compared to AT4. Our results followed the findings of Abdolmaleki and Heidarianpour, which showed training positively improved glucose tolerance and reduced IPGTT-glucose peak [45], but after 4 weeks of detraining, the positive effects vanished. This supports a continuous training program throughout life to maintain glucose homeostasis.

This study revealed that detraining for 4 weeks after 4 weeks of training increased the insulin level at T8 in AT4. When the training continued for a longer duration (8 weeks), an insulin level at T12 did not elevate. Suggesting there might be a cumulative effect of 8 weeks of moderate-intense aerobic training that prevents insulin increase after detraining. Omidi and Yousefi (2019) reported that fasting glucose and insulin significantly improved after 8 weeks of aerobic training [46]. On the other hand, exercise intervention reduced the fasting glucose but increased insulin concentration (*p* < 0.05) [47].

Interestingly, in the present study, the serum cholesterol level significantly decreased after 4 weeks of training (*p* < 10^−4^), which is consistent with previous results of Fahri et al. and Kraus et al. [48,49]. However, in AT4, after 4 weeks of detraining at T8, the cholesterol level increased by 36% compared to T4 (*p* < 0.25). Whereas in AT8, it was still significantly lower than baseline after 8 weeks of training and even after 4 weeks of detraining, showing that 8 weeks of training can limit the effects of detraining better than only 4 weeks of training. On the other hand, serum triglyceride levels decreased after detraining in AT4 (at T8 and T12, and AT8 at T12). However, the continuous training for 8 weeks has shown a significant increase in serum triglyceride levels compared to T0. This increase might be because of the increased energy requirement during exercise and the utilization of triglycerides as fuel. This is consistent with previous findings by Banz et al., who revealed that aerobic exercise training for 10 weeks increased triglycerides [50]. Contrarily, not all studies have found a significant alteration in lipid parameters after exercise training [51,52]. The present study reported a slight reduction in serum triglyceride levels after 4 weeks of detraining and even greater after 8 weeks. 

HDL is the most sensitive parameter to aerobic exercise compared to LDL and triglycerides. It is well-known that changes in HDL levels are more obvious in rats than other lipid parameters. It might be related to the greater sensitivity of HDL to training but also to the abundance of HDL in rats compared to the other lipid components [53]. The current study reported that serum HDL levels only increased after 8 weeks of training, indicating a dose effect of training. Nevertheless, after 4 weeks of training cessation at T12, the HDL level decreased by 23% compared to T8 (*p* < 0.05). Similarly, a reduction in HDL after 8 weeks of detraining (T12) was observed in AT4. Similar to our findings, LeMura et al. demonstrated that sixteen weeks of aerobic training significantly elevated blood HDL in young women (*p* < 0.05), but these alterations vanish after 6 weeks of detraining [54], while in our study, a significant reduction in serum HDL level can be seen after 4 weeks of detraining. In another study, HDL levels decreased after a brief interval of detraining [32], which is in accordance with our results. Moreover, in the current study, trained animals had lower LDL level, which was maintained when exercise training continued for 8 weeks. However, the favorable adaptation to a lower LDL level was lost following detraining. It was found that 4 weeks of detraining increased LDL level, but a greater increase was observed after 8 weeks. Notably, the LDL level increased after detraining but remained lower than the baseline concentration. This is consistent with the previous finding of Rogerio et al., who showed that detraining for 4 weeks after 8 weeks of training increased LDL concentration [55]. 

The liver is an important organ that helps to regulate glycemia under metabolic alteration during exercise by controlling gluconeogenesis and blood glucose production [56]. Our results showed a reduction in serum ALP levels after 4 weeks of training and even after detraining. Similarly, a previous study reported a 6.1% reduction in the ALP enzyme after 2 weeks of aerobic exercise in overweight and obese men [57]. Additionally, 8 weeks of training significantly increased serum ALT levels (*p* = 0.004) and to a greater extent after 4 weeks of detraining at T12 (*p* = 0.001) compared to baseline. Similarly, the ALT level increased abruptly after 4 weeks of detraining in AT4. This increase in ALT after training cessation might be associated with an increased risk of insulin resistance. Hanley et al. indicated that the increased serum ALT activity predicts the development of insulin resistance and type 2 diabetes in human populations, but without any apparent sign of liver injury [58]. The current training program failed to evoke a significant alteration in serum AST levels, which is in accordance with the previous study. A study on professional sportsmen found no significant difference in AST levels after the training intervention [59]. However, the AST level increased at T8 in AT4 and AT8 at T12 after 4 weeks of detraining. Previously, it was noted that an increase in AST activity is associated with insulin resistance, metabolic syndrome, and type 2 diabetes [60]. Given that detraining has a negative association with AST levels.

Hexokinase is an important glycolysis enzyme involved in skeletal muscle glucose metabolism. Acute stimulation of skeletal muscle, either by insulin or by contraction through exercise, increased hexokinase activity in human and rodent skeletal muscle [61,62,63]. Similarly, our result showed that 4 weeks of training increased hexokinase activity by 38% vs. baseline, and 8 weeks of training further increased by 13% vs. T4. Indeed, exercise training has a positive impact on hexokinase levels, but when the body switches to inactivity, all these positive adaptations could be lost. We observed that detraining for 4 weeks in AT4 at T8 reduced hexokinase by 60%, while in AT8 at T12, it reduced by 56%. It was reported that the decreased hexokinase II expression during physical inactivity could trigger an alteration similar to type 2 diabetes, such as altered insulin response and muscle glucose transport [64]. 

Additionally, glycogen is the polysaccharide storage form of glucose in skeletal muscle. The present study found that initially, glycogen content increased after 4 weeks of training at T4 compared to T0, but glycogen content decreased in the AT4 group after detraining at T8 and T12. Previously, training-induced reduction in muscle glycogen was reported as a key driver to the post-training improvement in insulin sensitivity [65,66]. Interestingly, the glycogen content declined when training continued for 8 weeks in the AT8 group, possibly due to greater skeletal muscle training adaptation. Low glycogen availability during training could shift to other substrates’ metabolism during and after exercise training [67,68]. However, after training cessation at T12 in the AT8 group, there was again a rise in glycogen content by 24% compared to AT4. Post-training skeletal muscle glycogen repletion might be influenced by hexokinase activity in the skeletal muscle, consistent with previous findings [69]. Evidence has shown that training significantly elevated the hexokinase II protein by 70%. In addition, an increase in muscle glycogen was not apparent after 1 day of training, but it increased by 40% after 4 days of training compared to pretraining [70]. 

Phosphofructokinase (PFK) is considered a rate-limiting enzyme when glucose enters glycolysis pathways and is allosterically activated by ADP, AMP, and Pi. The present study did not observe any significant change in PFK in either group during the training and detraining period. Our results are similar to the previous findings of Henriksson and Reitman, which demonstrated that training for 7–8 weeks did not cause alteration in phosphofructokinase activity in human skeletal muscle [71]. Unlike hexokinase, PFK was maintained during the training and detraining, suggesting that glucose may not have entered glycolysis pathways. This leads to the body’s utilization of other energy sources like fats (triglycerides). For instance, fat is an important substrate for muscle contraction at rest and during exercise [72,73].

We observed an increase in serum triglyceride levels after training. Circulating triglycerides in the bloodstream could be broken down by an enzyme named lipoprotein lipase (LPL). These triglycerides are embedded in very low-density lipoproteins (VLDL) and chylomicrons traveling through the bloodstream [74]. LPL is also known to regulate lipoprotein and may reduce LDL [75]. LPL protein expression declined in AT4 after training cessation, which aligns with a previous study on rats that demonstrated that inactivity from 11 h to 11 days reduced LPL activity by 80%, indicating that physical inactivity may reduce muscle fat uptake [76]. In addition, adipose triglyceride lipase (ATGL) catalyzes adipose triglyceride and mobilizes lipids for energy production. ATGL also controls lipid homeostasis in other tissues [77]. We observed that 8 weeks of training increased protein expression of LPL by 6-fold and ATGL by 20-fold in the muscle, which is in line with former studies illustrating that regular endurance training results in greater muscle ATGL and LPL contents [78,79,80]. The increase in ATGL protein expression could maintain the low metabolic concentration of muscle fatty acids, thus improving insulin sensitivity [81]. The reduction in the expression of LPL and AGTL after detraining may reduce the catabolism of LDL; therefore, the LDL level increased during the detraining period. However, no significant change was found in fatty acid transporter FATP4 expression.

Training for 4 weeks slightly increased PFK and pyruvate, but further elevation was not observed with 8 weeks of training. Indeed, PFK is the rate-limiting enzyme in the glycolytic pathway; no change in PFK leads to the fact that the body has utilized other substrates to fulfill the energy requirement during exercise. Further, 4-week training increased glycogen content, which might be influenced by an increased hexokinase activity. However, when training continued for 8 weeks, the glycogen content decreased, while there was an increase in triglycerides level and protein expression of LPL and ATGL, suggesting that there might be a shift towards utilizing other substrate metabolisms when training continues for 8 weeks. The body might have started utilizing fat as fuel rather than carbohydrates at this stage. Additionally, training-induced elevated LPL expression may contribute to increased triglyceride-rich lipoprotein catabolism, thus reducing LDL levels. Thus, alterations in substrate use may contribute to the beneficial effect of regular exercise training to reduce the risk of obesity, insulin resistance, and type 2 diabetes.

Contrarily, we observed that training cessation after 4 weeks of training results in a loss of adaptations. For instance, training cessation reduced glucose tolerance, hexokinase activity, glycogen content, and protein expression (LPL and ATGL). Training cessation reduced LPL expression, which could reduce the catabolism of triglyceride and LDL. While detraining increased serum insulin, total cholesterol, LDL, and liver enzymes (ALT and AST) levels. All of these alterations have a negative impact on the metabolic function of the body and might be an indicator for the development of impaired glucose tolerance, insulin resistance, obesity, and eventually type 2 diabetes. 

Notably, the increasing prevalence of type 2 diabetes and insulin resistance is not due to environmental disruption of genes, which is a common misconception. In fact, physical inactivity is an abnormal event for a genome programmed for physical activity. Therefore, insufficient physical activity fails to regulate normal biochemical and molecular events in maintaining glucose metabolism. Considering that the human body is designed to be physically active, the transition from a physically active lifestyle to an inactive lifestyle may have induced some metabolic changes, which is supported by the results of this study. Our study offers new insights by considering physically trained animals as controls for analyzing the downstream pathways involved in glucose metabolism. The current findings may have significant implications for preventing and treating diabetes, which is responsible for 6.7 million deaths (one in every five seconds), and the healthcare cost was about USD 966 billion in 2021 [82]. Therefore, it is necessary to promote regular physical activity, an innate behavior adopted by the human genome. Further research must be conducted to explore the alterations in metabolic pathways when the body switches from physical training to detraining. It is also required to identify the alteration in “active” genes that result in disease development when exposed to sedentary conditions.

There are some limitations in this study. Although a treadmill test was performed on each animal to assess their physical fitness through lactate release to individualize their training, it was challenging to apply specific training adapted to each animal. This is partially due to the equipment, which had six lanes but only a one-speed controller for all lanes. Another limitation is that only two detraining durations were selected. It is important to analyze different detraining durations to identify at exactly which time point the body starts losing the metabolic adaptations induced by exercise training.

## 5. Conclusions

This study identified the impact of training and detraining on metabolic adaptation, glucose metabolism, and the dose effect of training and detraining. Regular physical activity has improved glucose, insulin, lipid profile, liver enzymes, and glucose metabolism. A significant improvement was seen in the AUC for IPGTT, and the positive effect on glucose metabolism was lost during the detraining period. Further, no significant change was seen in HDL level after 4 weeks of training, but there was a significant elevation in HDL level at T8, indicating a dose effect of training. Training elicits a significant reduction in serum total cholesterol, LDL level, and ALP (*p* < 0.05) and an increased ALT level. Training increased hexokinase activity, but no change was seen in PFK. However, detraining increased glycogen recovery and pyruvate content after 8 weeks of training. Further, 8 weeks of training increased triglyceride levels, and enzymes (LPL, ATGL) were observed; the body lost this adaptation after 4 weeks of detraining. Further, detraining after 4 weeks of training immediately caused a loss of adaptations, thus resulting in the reduction of AUC of the glucose tolerance, hexokinase activity, glycogen content, and increased insulin level, total cholesterol, LDL level, and liver enzymes (ALT and AST). Conclusively, 4 weeks of daily physical training has shown beneficial effects in regulating glucose metabolism, with greater benefits associated with a longer training duration. Four weeks of detraining only were enough to start losing these positive adaptations. These results highlight the deleterious effects of physical inactivity and the importance of daily and constant endurance activity at moderate intensity to prevent the development of metabolic abnormalities likely to lead to complications such as type 2 diabetes. 

## Figures and Tables

**Table 1:** Weekly body weight, feed intake, and blood glucose level (Mean ± S.D.).

		T0	T4	T8	T12
Body Weight	AT4	170.03 ± 20.80	258.05 ± 17.34 a	324.30 ± 36.34 ab	351.40 ± 46.86 ab
	AT8	173.77 ± 27.71	258.25 ± 13.63 a	330.16 ± 22.26 ab	356.57 ± 8.86 ab
Feed Intake	AT4	24.35 ± 3.18	25.86 ± 4.47	23.73 ± 2.30	21.41 ± 1.33
	AT8	24.35 ± 3.18	25.86 ± 4.47	23.39 ± 4.43	23.33 ± 1.85

Aerobic training of 4 weeks (AT4), aerobic training of 8 weeks (AT8). Body weight and feed intake was measured in grams (g). a Statistically significant difference with T0. b Statistically significant difference with T4.

## Data Availability

The datasets generated and analyzed during the current study are available from the corresponding authors upon reasonable request.

## Abbreviations

Intraperitoneal glucose tolerance test (IPGTT), pyruvate, glycogen, protein expression of lipoprotein lipase (LPL), adipose triglycerides lipase (ATGL), and fatty acid transport protein 4 (FATP4).
